# Direct Evidence for Excitation Energy Transfer Limitations Imposed by Low-Energy Chlorophylls in Photosystem I–Light Harvesting Complex I of Land Plants

**DOI:** 10.1021/acs.jpcb.1c01498

**Published:** 2021-03-31

**Authors:** Mattia Russo, Anna Paola Casazza, Giulio Cerullo, Stefano Santabarbara, Margherita Maiuri

**Affiliations:** † Istituto di Fotonica e Nanotecnologie del Consiglio Nazionale delle Ricerche, Dipartimento di Fisica, Politecnico di Milano, Piazza Leonardo da Vinci 32, 20133 Milano, Italy; ‡ Istituto di Biologia e Biotecnologia Agraria, Consiglio Nazionale delle Ricerche, Via Bassini 15a, 20133 Milano, Italy; § Photosynthesis Research Unit, Centro Studi sulla Biologia Cellulare e Molecolare delle Piante, Consiglio Nazionale delle Ricerche, Via Celoria 26, 20133 Milano, Italy

## Abstract

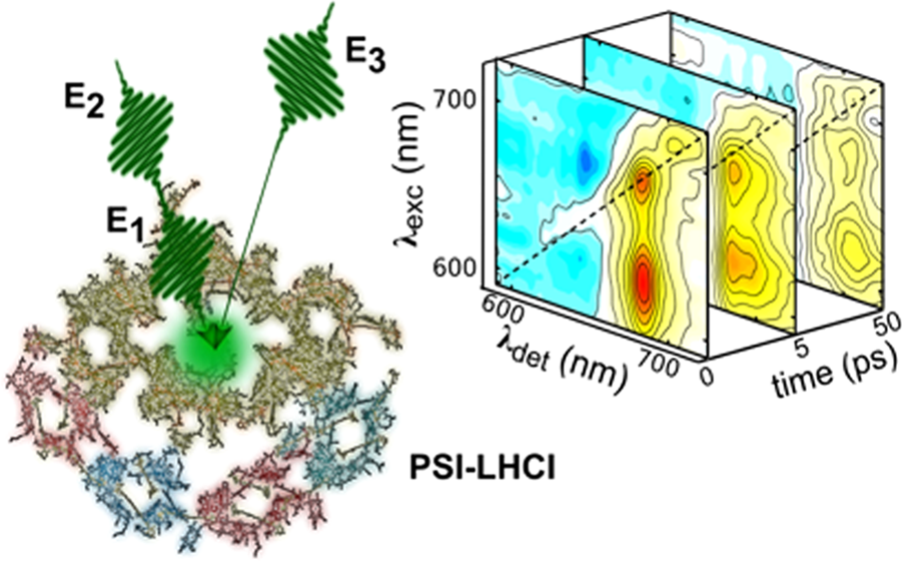

The overall efficiency of photosynthetic energy conversion depends both on photochemical and excitation energy transfer processes from extended light-harvesting antenna networks. Understanding the trade-offs between increase in the antenna cross section and bandwidth and photochemical conversion efficiency is of central importance both from a biological perspective and for the design of biomimetic artificial photosynthetic complexes. Here, we employ two-dimensional electronic spectroscopy to spectrally resolve the excitation energy transfer dynamics and directly correlate them with the initial site of excitation in photosystem I–light harvesting complex I (PSI-LHCI) supercomplex of land plants, which has both a large antenna dimension and a wide optical bandwidth extending to energies lower than the peak of the reaction center chlorophylls. Upon preferential excitation of the low-energy chlorophylls (red forms), the average relaxation time in the bulk supercomplex increases by a factor of 2–3 with respect to unselective excitation at higher photon energies. This slowdown is interpreted in terms of an excitation energy transfer limitation from low-energy chlorophyll forms in the PSI-LHCI. These results aid in defining the optimum balance between the extension of the antenna bandwidth to the near-infrared region, which increases light-harvesting capacity, and high photoconversion quantum efficiency.

## Introduction

Photosystem I (PSI) is one of the two pigment–protein supercomplexes essential for oxygenic photosynthesis, which mediate the light-driven electron transport from water to NADP^+^. In land plants, PSI is organized into two functional and structural moieties: the core complex, harboring the reaction center (RC), where charge separation and successive electron transfer reactions occur, and an external antenna complex, collectively referred to as light harvesting complex I (LHCI). The PSI-LHCI supercomplex is considered as one of the best performing photochemical machineries known in nature, showing the highest efficiency among other photosystems, approaching unity.^1−3^

This very high photon conversion yield is maintained even though some light-harvesting antenna pigments absorb at lower energies than the RC chlorophylls (Chl), implying a thermodynamically unfavorable uphill excitation energy transfer (EET) to the photocatalytic site.^4^ In higher plants, these low-energy absorbing chromophores, often referred to as “red forms”, are mainly located in the LHCI antenna,^4−6^ and are therefore, from a structural perspective, located at the periphery of the complex.^7^ Moreover, more than one low-energy state is present in the LHCI, and each of the two dimers (Lhca1/Lhca4 and Lhca2/Lhca3) comprising it appears to bind a long-wavelength spectral form emitting at ∼730/735 nm.^8^ This scenario is different from the case of cyanobacterial PSI, where the red forms, whose exact characteristics are species-dependent,^9^ are instead located in the core complex, and hence spatially closer to the RC.

Since EET processes temporally overlap with the primary photochemical charge separation event and the first charge stabilization step, the direct and independent observation of these processes is challenging. The problem is further complicated by spectral congestion, since a large pigment–protein complex like PSI-LHCI binds, in total, over 150 Chls. As a result, the interpretation of the data often needs to rely on kinetic modeling. Thus, although both in the PSI of cyanobacteria^9−11^ and in the PSI-LHCI supercomplex of higher plants^4,12,13^ it has been proposed that uphill EET can limit the effective photochemical trapping time, the extent of this limitation remains to be unambiguously determined, at least for the case of supercomplexes, in which the red forms are located in the external antenna.

From a biological perspective, the physiological role of red forms appears to be dominant under shading conditions, when photon fluxes are low and therefore the photosynthetic process is limited by light absorption and hence by the photon conversion efficiency.^14^ It is moreover relevant to the understanding of photochemical processes in cyanobacteria that incorporate the intrinsically red-shifted Chl*f* in their antenna^15−17^ and possibly also in the RC.^18^ Furthermore, understanding the trade-off between the antenna bandwidth and the energy of the photocatalytic site can be of key importance for the design of broad-band artificial photosynthetic molecules and/or devices.

The most straightforward approach to test experimentally the limitations imposed by antenna red forms on EET and photochemical trapping processes is to perform experiments in which these forms are directly excited. Although some experimental evidences have been collected in cyanobacterial PSI, which consists of the core complex only, initially by time-resolved fluorescence^10,11^ and successively by ultrafast transient absorption (TA)^19,20^ and two-dimensional electronic spectroscopy (2DES),^21,22^ limited direct information is available for the large PSI-LHCI supercomplex of higher plants.^23^

2DES is particularly powerful as it offers the possibility of directly test and cross-correlate the frequency of the initial site of excitation with the observed dynamics at a specific detection frequency on an ultrafast timescale. Recently, Akhtar et al.^23^ studied both the core complex and the PSI-LHCI supercomplex of land plants using 2DES, but the excitation pulse bandwidth allowed only partial coverage of the long-wavelength absorption tail, up to ∼705 nm, therefore only partially covering the red forms absorption. Here, we investigate the excited-state dynamics in the PSI-LHCI supercomplex isolated from spinach by 2DES using ultra-broad-band pulses covering the 580–725 nm range, that allows us to explore direct red forms excitation in further detail. The complex was studied under both conditions of “open” and “closed” RCs (the latter achieved by chemical oxidation of the terminal electron donor *P*_700_), thereby comparing EET in the presence and absence of photochemical quenching.

## Methods

### Sample Preparation

PSI-LHCI was purified from unstacked spinach thylakoids as previously described.^13^ The membranes were solubilized at 1 mg/mL Chl with 1% w/v β-dodecyl maltoside, which also replaced octylglucopyranoside in the successive sucrose density gradients. The PSI band from the sucrose gradient was concentrated by spinning at 180 000*g* for 2 h and finally resuspended in 25 mM tricine, 5 mM MgCl_2_, and 0.003 w/v β-dodecyl maltoside. The sample at a concentration equivalent to 75 OD cm^–1^ was placed in a 200 μm thick flow cuvette, and incubated with 30 mM sodium ascorbate and 150 μM *N*,*N*,*N*′,*N*′-tetramethyl-*p*-phenylenediamine (TMPD) (open-center conditions) or 30 mM K_3_Fe(CN)_6_ (closed-center conditions).

### Two-Dimensional Electronic Spectroscopy

2DES simultaneously meets the requirements of high temporal and spectral resolution, exploiting the generation of the third-order nonlinear response function of the system after the interaction with three properly delayed broad-band laser pulses. Here, we adopted the partially collinear geometry in which the first two collinear phase-locked pulses delayed by a time *t*_1_ (coherence time), generated by a birefringent interferometer, act as a pump. The third pulse acts as a probe and is delayed by the time *t*_2_ (population or waiting time) with respect to the second pulse.^24^ By acquiring the signal as a function of coherence time *t*_1_ for a fixed population time *t*_2_ and performing a Fourier transform with respect to *t*_1_, one obtains two-dimensional excitation/detection correlation maps.

The ultra-broad-band visible pulses were generated by a noncollinear optical parametric amplifier in the visible and temporally compressed with a pair of chirped mirrors to reach sub-20-fs duration. The polarizations of the excitation and detection pulses were orthogonal to reduce the contribution of scattered light, which is almost unavoidable due to the dimension of the PSI-LHCI supercomplex. The adopted fluence was 28 μJ/cm^2^, low enough to avoid singlet–singlet annihilation. The combination of high temporal resolution afforded by the broad-band laser pulses and high excitation/emission frequency resolution makes 2DES a perfect tool to study ultrafast processes in photosynthetic complexes. Experimental data were complemented by a global fitting procedure of the extended 2DES datasets.^25^ Fitting of the early time dynamics, which requires considering the instrumental response function (IRF) of the system, was performed by convolving a multiexponential response with the IRF, given by the cross-correlation of the pump and probe pulses.

## Results and Discussion

**Figure 1:**
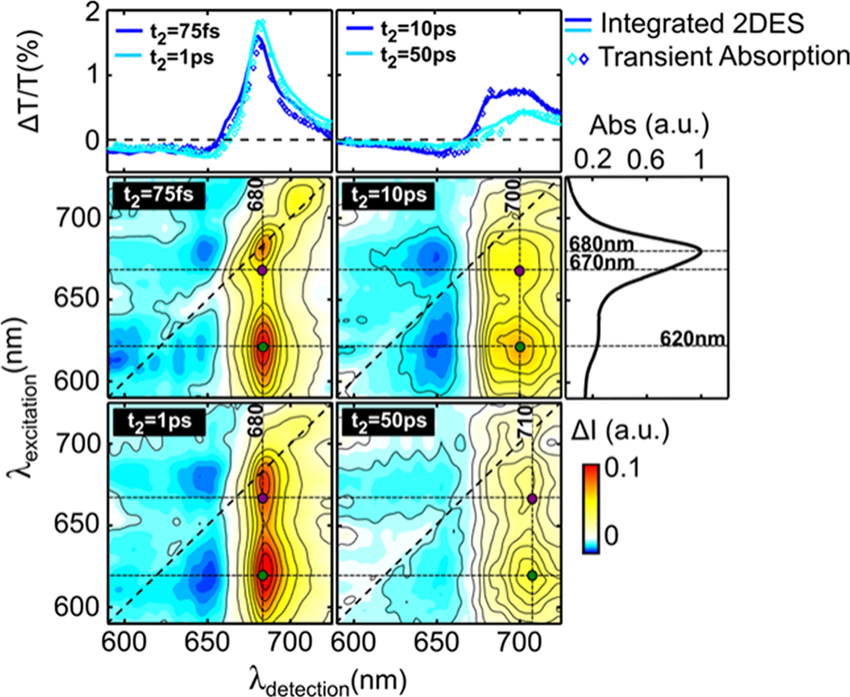
2DES maps of the PSI-LHCI supercomplex under open-center conditions at four different waiting times: *t*_2_ = 75 fs, 1 ps, 10 ps, and 50 ps. (Top) 2DES maps integrated along the excitation wavelength axis (solid lines) overlapped with the TA spectra (symbols). (Right) Portion of the steady-state absorption spectrum from 590 to 730 nm. Filled dots identify selected cross-peaks discussed in the text.

Figure 1 shows four 2DES maps for PSI-LHCI under open-center conditions at waiting times *t*_2_ = 75 fs, 1 ps, 10 ps, and 50 ps along with the steady-state absorption spectrum on the right and the excitation wavelength integrated 2DES spectra overlapped with the TA spectra on top. 2DES maps at *t*_2_ = 75 fs and 1 ps show an intense diagonal peak at 680/680 nm excitation/detection wavelength, corresponding to the ground-state bleaching (GSB) of the Q*_y_* transition of Chl*a* spectral forms responsible for the main absorption band (the so-called “bulk” Chls). The diagonal peak is accompanied by several off-diagonal cross-peaks,^26^ indicating both coupling and EET between the Q*_y_* transition of bulk Chl*a* antenna chromophores and the shorter-wavelength Q*_x_* band of Chl*a*, as well as both Q*_y_* and Q*_x_* bands of Chl*b*.

Intramolecular EET processes between the 0–0 (Q*_y_*) transition and the coupled vibrational modes are however difficult to disentangle from intermolecular EET even by 2DES because of extensive spectral congestion, due to the large number of chromophores in the system, and because of inhomogeneous and homogeneous broadening of the transition energies. Yet it is worth noting that intramolecular EET, which is mediated by vibrational coupling, tends to localize the excitation on the diagonal of 2DES maps at early delays, whereas for excitation at wavelengths shorter than ∼675 nm, most of the intensity lies in the off-diagonal cross-peaks, indicating the occurrence of ultrafast EET from the short-wavelength spectral forms to the dominant absorption centered around 680 nm. The maps also present a broad and weak negative signal that corresponds to excited-state absorption (ESA) of the bulk Chls. The presence of ESA contributions might also mask the direct observation of vibronic coupling, as most of the partially resolved Chl vibronic progression falls in the same spectral window.

Moreover, a slight broadening of the main diagonal peak toward long wavelengths is detected already at *t*_2_ = 75 fs and becomes more obvious at *t*_2_ = 10 ps, when new cross-peaks centered around 700 nm are detected, close to the absorption peak of the electron donor *P*_700_, which is an integral part of the RC. Further spectral evolution is observed at *t*_2_ = 50 ps, when the position of the cross-peak shifts further to the red from 700 nm, i.e., into the spectral window dominated by Chl red forms.^4,12,27−29^ This shift is mainly due to the relaxation of the residual GSB contribution from the bulk and moderately red-shifted Chl spectral forms, as the signal intensity in the long-wavelength tail is almost the same at *t*_2_ = 10 and 50 ps (see the top right panel of Figure 1). Notably, at *t*_2_ = 50 ps, the 2DES map still shows significant intensity at 700 nm, consistent with the photochemical population of the long-lived *P*_700_^+^ cation and demonstrating thereby the open state of the RCs in the complex. To gain further detail and decouple pure EET processes from the population of a long-lived radical pair following photochemical trapping, the 2DES experiments were also performed under closed-RC conditions, when *P*_700_ is preoxidized by ferricyanide. The corresponding maps are reported in Figure S2 in the Supporting Information (SI).

**Figure 2:**
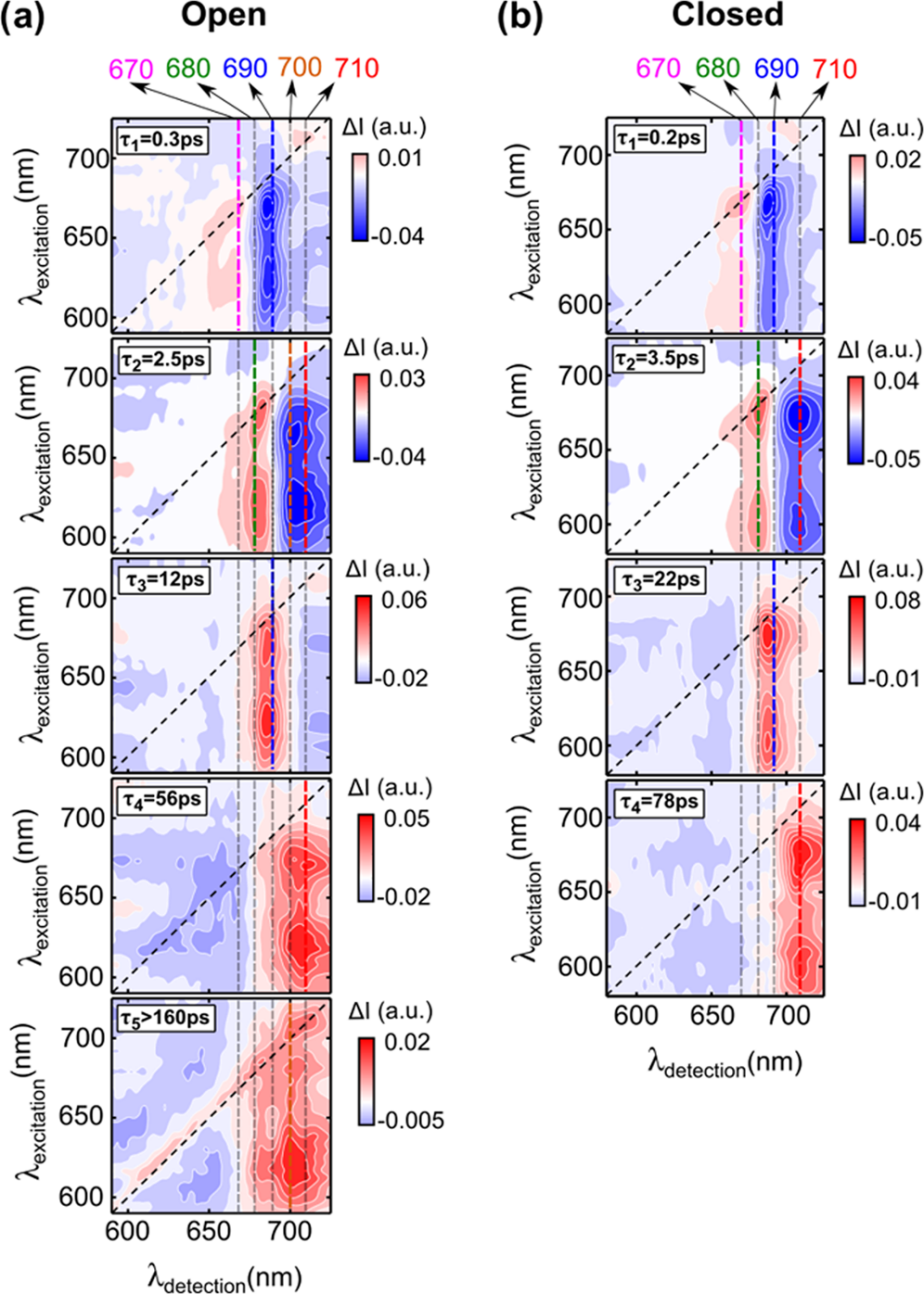
Global analysis results: (a) 2D-DAS maps of PSI-LHCI with open RCs. The temporal evolution of the system can be described with five time constants: 0.3, 2.5, 12, 56 ps, and a nondecaying component (limited by the maximum delay acquired for the measurement); (b) 2D-DAS maps of PSI-LHCI with closed RCs. Note the different colorbar scales for the open- and closed-RC configurations. The temporal evolution of the system can be described with four time constants: 0.2, 3.5, 22, and 78 ps. Slices of the 2D-DAS maps at representative wavelengths are presented in Figures S4 and S5 for open and closed centers, respectively.

The 2DES maps acquired under both open- and closed-center conditions were decomposed by applying a global analysis algorithm to the data. This approach allows us to model the temporal evolution of the entire 2DES dataset with a series of exponential decays. The results of this analysis are expressed by two-dimensional decay associated spectra (2D-DAS) that are energy correlation maps showing the amplitude of a specific exponential decay for every point of the 2DES map.^25^ The sign of the amplitude in the 2D-DAS allows one to identify formation or decay signals; specifically, a positive amplitude represents a positive exponential decay, meaning that GSB signals are decaying, and a negative amplitude identifies a negative exponential decay corresponding to GSB formation or ESA decay. Figure 2a reports the 2D-DAS for the PSI-LHCI supercomplex in open-RC conditions, corresponding to lifetimes of 0.3, 2.5, 12, 56, and >160 ps. The 2D-DAS of the PSI-LHCI supercomplex under closed-RC conditions are shown in Figure 2b and correspond to lifetimes of 0.2, 3.5, 22, and 78 ps. These values are in very good agreement with those previously reported for TA on the same sample^13^ but recorded only for a limited number of pump wavelengths, all shorter than the RC resonance and with overall lower temporal resolution. Furthermore, they are rather close to the values observed in PSI-LHCI isolated from different plants^9,12,27−31^ or from green algae^32−35^ and compatible with the PSI core of cyanobacteria.^35−37^

The overall excited-state dynamics are not largely affected by the redox state of *P*_700_ (Figure 2b), in agreement with previous reports.^38−40^ The additional long-lived component (>160 ps) under open-center conditions accounts for the contribution of *P*_700_^+^ whose decay is several orders of magnitude slower than the timescales of EET and primary electron transfer events. The longest-lived component under open-center conditions contains, however, also contributions from the red forms, as observable from the signal along the diagonal at wavelengths longer than 700 nm, which correspond to the ∼80 ps retrieved for closed-center conditions.

The 2D-DAS highlight the presence of two kinetic components of excited-state equilibration, one occurring on the subpicosecond timescale, and involving transfer to the bulk Chl forms, having a GSB maximum in correspondence to the 680 nm steady-state absorption peak, and a second phase, characterized by a lifetime of ca. 3–4 ps, corresponding to transfer to the low-energy forms, present both in the antenna and in the RC, considering that the absorption of *P*_700_ is in any case red-shifted with respect to the bulk of the antenna. The successive components are largely dominated by relaxation of the partially equilibrated state of the supercomplex, which is however faster for the bulk than for the long-wavelength states. Thus, from the inspection of the 2DES maps at selected delays and the 2D-DAS it is immediately appreciable that, when pumping at wavelengths shorter than ∼700 nm, the excited-state dynamics show very little dependence on the initial site of excitation. Interestingly, this also concerns preferential excitation of Chl*b* in the 630–650 nm window, where the relative contribution of this pigment is expected to be more relevant. For excitation at wavelengths shorter than the RC Chls absorption, the 0.3 ps component is dominated by EET to the bulk Chls; the 2.5–3.5 ps component contains contributions from slower equilibration with the low-energy antenna states, including those in the RC, as well as population of the primary radical pair;^12,23,31,34^ the 10–20 ps component represents the main deexcitation of the bulk Chls, due to charge stabilization, and contains contributions also from the relaxation of a subpopulation of whole of red forms pools, whereas the slowly decaying component includes both the relaxation of the lowest-energy forms in the antenna and, under open-center conditions, also the signal due to the long-lived, stabilized, radical pair.

This general behavior, which accounts for excitation at wavelengths shorter than 700 nm, displays significant differences upon long-wavelength pumping. Despite the lifetimes being the same, the relative amplitudes, and therefore the overall excited-state dynamics, is distinct upon direct red forms excitation, as notable from the intensity and sign of the off-diagonal amplitudes in the 2D-DAS maps. A zoomed view of the 2D-DAS maps centered on the red-form absorption window is presented in Figure S3 of the SI.

**Figure 3:**
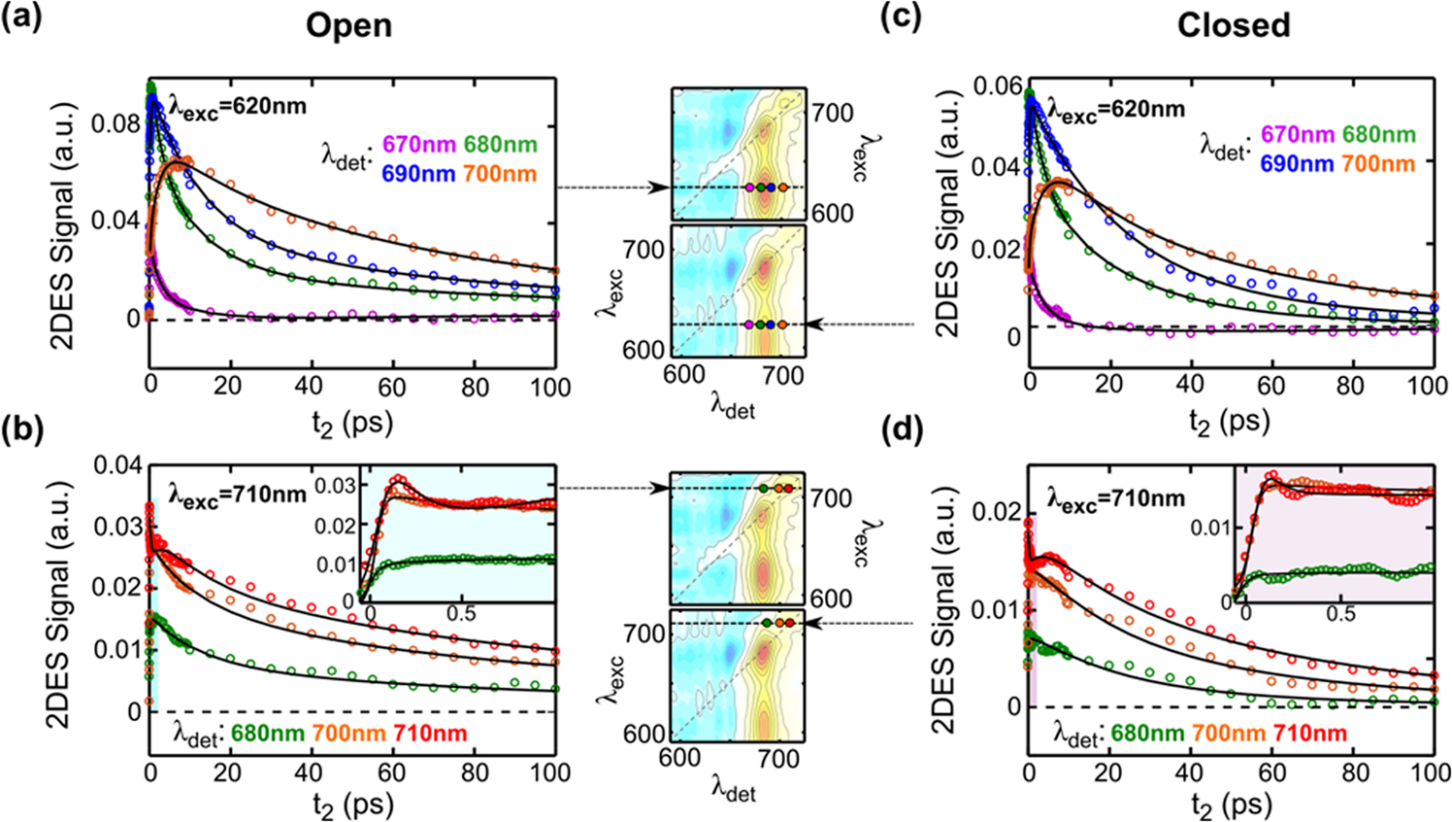
*t*_2_ Time traces from the 2DES data of PSI-LHCI in (a) open- and (c) closed-center conditions obtained by exciting at 620 nm and probing at 670 nm (violet), 680 nm (green), 690 nm (blue), and 700 nm (orange). *t*_2_ Time traces from the 2DES data of PSI-LHCI in (b) open and (d) closed configurations obtained by exciting at 710 nm (Chls red forms) and probing at 680 nm (green), 700 nm (orange), and 710 nm (red). In all panels, the dots represent the experimental data, and the continuous black lines are the fits obtained from the global analysis. The insets show a zoom on the kinetics up to 1 ps in which the best fit is obtained by convolution of the IRF of the system with multiexponential decays. For the open (closed) configuration, we used five (four) decays: τ_1_–τ_5_, where τ_3_–τ_5_ are fixed to the values obtained from the global analysis. The results are reported here. For λ_probe_ = 680 nm: τ_1_ = 0.3 ps (0.3 ps), τ_2_ = 2 ps (5 ps); for λ_probe_ = 700 nm: τ_1_ = 0.35 ps (0.3 ps), τ_2_ = 5 ps (3.5 ps); for λ_probe_ = 710 nm: τ_1_ = 0.2 ps (0.4 ps), τ_2_ = 5 ps (4 ps). The fits have an uncertainty of ±0.05 ps.

This is also clearly illustrated in Figure 3 by the comparison of the kinetics (as a function of *t*_2_) obtained by exciting the PSI-LHCI with open (Figure 3a,b) and closed RCs (Figure 3c,d). The kinetics at early times and the corresponding fits are reported in the insets of Figure 3b,d. Under open-RC conditions, upon unselective excitation at 620 nm (Figure 3a), mainly corresponding to the absorption peak of the Q*_x_* band of Chl*a*, the kinetics monitored close to the absorption maximum (680 nm), after a very fast rise, decay promptly to the residual long-lived signal. This is due to both the nondecaying contribution of *P*_700_^+^ and to the slowest relaxation component from the red-most spectral forms. On the other hand, when the kinetics are monitored at 700 nm, which is a marker band for *P*_700_^+^ at open centers, a pronounced rise is observed, followed by a slow decay.

Upon excitation at 710 nm (Figure 3b), the observed dynamics change drastically. Even though at this wavelength the red forms are expected to be predominantly excited, partial excitation of the RC Chls is also expected because of the broad absorption bandwidth of *P*_700_. The GSB signal of bulk Chls probed at 680 nm is characterized by a clear rise (a zoomed decay is highlighted in the inset of Figure 3b, green curve), indicating an uphill EET, whereas when monitoring either the RC Chls at 700 nm or the red forms at 710 nm (orange and red curves, respectively, in the inset of Figure 3b), a small but prompt decay to a nearly stationary level is observed. The fast relaxation (350 and 200 fs, respectively, for these two traces) can be interpreted as a contribution from EET of these moderately red-shifted forms to the more red-shifted states, as well as from the uphill EET to the bulk Chls.

**Figure 4:**
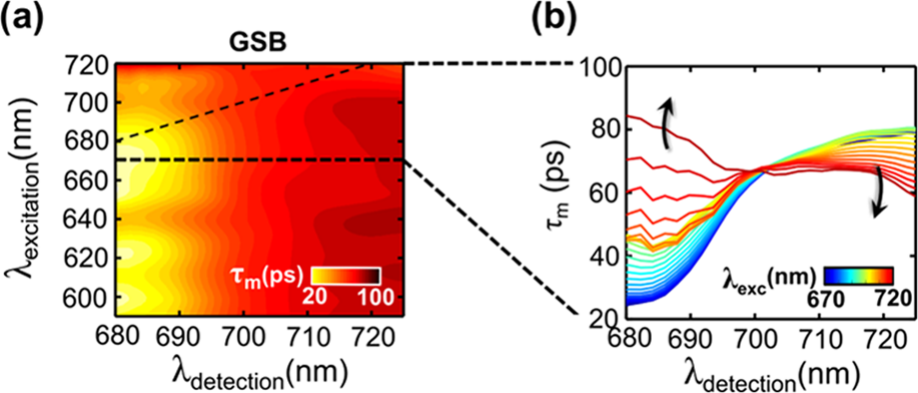
(a) τ_m_ Map derived from the 2D-DAS of Figure 2b for PSI-LHCI under closed-center conditions, in the GSB-dominated spectral window. (b) Slices showing the wavelength dependence of τ_m_ at selected pump wavelengths.

The difference in the overall dynamics, depending on the site of excitation, can be compactly visualized by contour plots of the first moment of the decay distribution, τ_m_ (defined as τ_m_(λ_1_,λ_2_) = ∑_*i*_*A*_*i*_(λ_1_,λ_2_)·τ_*i*_^2^/∑_*i*_*A*_*i*_(λ_1_,λ_2_)·τ_*i*_, where *A*_*i*_(λ_1_,λ_2_) are the 2D-DAS and τ_*i*_ are the corresponding lifetimes; see also Section IV of the SI for further details). The application of decay moments is common practice in the analysis of time-resolved fluorescence data.^39^ However, despite the generality of the approach, it has been seldom applied to TA experiments, and it is here nonetheless extended to the analysis of 2DES data. Figure 4 shows the τ_m_ contour map (Figure 4a) together with slices at selected excitation wavelengths, calculated for closed-center conditions for pump wavelengths >670 nm (Figure 4b). For open centers, the estimation of τ_m_ is made more cumbersome by the presence of the nondecaying *P*_700_^+^ contribution, which needs to be fully deconvoluted to avoid distortions in the parameter estimation. Nonetheless, due to the observed similarity of the relaxation for open and closed centers, we consider the information obtained from the latter as significant also for fully photochemically active conditions. The observed rapid excited-state decay at closed centers is due to either a direct quenching by *P*_700_^+^ or, more likely, to nonradiative deexcitation to the ground state of a photochemically populated radical pair which cannot be further stabilized when *P*_700_ is already oxidized.^40,41^ Therefore, it can be considered as a “pseudo-photochemical” quenching process. In both cases, any EET migration limitation from the antenna to RC would remain even at closed centers.

The contour map of τ_m_ for the GSB-dominated region (Figure 4a) shows that, for excitation at wavelengths shorter than 700 nm, the first moment of the decay kinetics increases smoothly from short to long detection wavelengths. This is in analogy to what observed previously in fluorescence lifetime studies but calculating the average lifetimes, τ_av_, defined as τ_av_(λ_1_) = ∑_*i*_*A*_*i*_(λ_1_)·τ_*i*_/∑_*i*_*A*_*i*_(λ_1_), that corresponds to an amplitude-weighted zero-order moment of the decay distribution^4,28,29,42^ (see also the SI for a brief discussion). The smooth increase of τ_m_ toward long wavelengths can be interpreted in terms of a partial energy diffusion barrier associated with equilibration on the antenna red forms. This behavior appears slightly more pronounced for excitation in the 630–645 nm window, which is where Chl*b* (Q*_y_*) absorbs predominantly (see Figure S6 in the SI). Since Chl*b* is bound to the LHCI complexes that also harbor the red forms, the slightly more pronounced increase in τ_m_ is interpretable in terms of slower energy equilibration between the external antenna complexes, due to preferential coupling with the low-energy Chls, rather than in terms of slow EET from Chl*b* spectral forms themselves. On the other hand, the trend observed is remarkably different upon direct excitation of the red forms at wavelengths longer than 700 nm. Their direct relaxation becomes somewhat faster because of the prompt depopulation of these states associated with uphill EET to shorter-wavelength-absorbing pigments. Most significantly, the value of τ_m_ for the bulk Chls increases considerably when exciting above ∼700 nm. The progressive slowdown of the excited-state dynamics upon excitation at longer wavelengths is evidenced by the selected slices of the contour map reported in Figure 4b.

The direct correlation between excitation and detection wavelength provided by 2DES demonstrates unambiguously the impact of low-energy Chls red forms on the overall deexcitation dynamics in PSI. Our analysis performed on systems with closed centers can be reasonably extended to the open-center case. The slowing down of the dynamics upon excitation of the red forms is sizable for the bulk Chls, with a progressive lengthening of the mean decay lifetime from a factor of ≈2, when exciting at 700 nm, to a factor of ≈3 in the 705–715 nm pump wavelength window. Nevertheless, the dynamics remain relatively rapid, as the highest value of τ_m_ is ≈80–90 ps, which is over 20 times faster than the natural lifetime of Chls in the absence of photochemical and/or dissipative processes (∼2 ns). Thus, although the presence of antenna states red-shifted by 20–30 nm with respect to the RC Chls negatively affects the EET dynamics and, consequently, the overall trapping dynamics, the photon utilization efficiency remains close to unity.

## Conclusions

PSI is an intriguing system from the photochemical perspective, because it operates with an extremely high photon conversion quantum efficiency, approaching unity, even in the presence of energy states in its antenna network that absorb at lower energy than the chromophore involved in photochemical conversion. Thermodynamic reasoning would imply a competition for localization of the excited states between the RC and the low-energy Chl red forms, which should then decrease the photon conversion quantum efficiency. Although limitations imposed by EET have been previously observed by employing 2DES for the cyanobacterial core system, which has an antenna network dimension of ∼80 Chl/RC,^9−11,19,20^ we have here demonstrated a significant effect of low-energy Chls on the overall trapping/EET dynamics even in the larger (∼150 Chl) RC of higher plants, where the red forms are located much further at the periphery of the supercomplex. The extent of EET slowing down correlates with the specific energy of the red form, being more pronounced for the red-most forms, which have been attributed to Chls bound to the Lhca3 and Lhca4 complexes.^6,8,43−45^ At the same time, EET from the high-energy absorbing chromophore Chl*b*, which is also bound to LHCI, and that can be simultaneously investigated by broad-band 2DES, is less pronounced. This seems to indicate that the slower EET is not due to slow Chl*b* to Chl*a* EET, but rather to the co-localization of Chl*b* and the red forms in the LHCI complexes.

These results allow us to conclude that it is the energy level of the chromophores, rather than their specific location within the photosystem with respect to the RC, which contributes the most to their energetic equilibration dynamics. This is a relevant piece of information for defining within a general framework the balance between increase of antenna bandwidth and optimization of quantum conversion efficiency in complex chromophore networks comprising molecules absorbing at longer wavelengths than the site of photochemical energy conversion. Our conclusions are relevant both to biology, where red forms are widespread but strongly organism-dependent,^46^ and to the *de novo* design of artificial light-harvesting materials.

## Supporting Information Available

Section I: general structure of the PSI-LHCI supercomplex and superimposition of the laser bandwidth used in 2DES measurements on the steady-state absorption spectrum; Section II: 2DES maps for PSI-LHCI at closed-center conditions; Section III: expanded 2DES maps at representative *t*_2_ delays and 2D-DAS focused on the red-form-dominated spectral window. Also shown in this section are the DAS at selected pump wavelengths, sliced from the 2D-DAS maps of Figure 3 to allow direct comparison with classic TA measurements; Section IV: comparison of τ_m_ slices across the pump axis (from Figure 4) with TC-SPC results from previous studies; Section V: reproducibility of the results and analysis; and an independent dataset, acquired under similar experimental conditions is presented to support the reproducibility of all of the main spectra and kinetic features reported in the main text (PDF)

## Abbreviations

PSI-LHCI: photosystem I–light harvesting complex I
RC: reaction center
EET: excitation energy transfer
Chl: chlorophyll
TA: transient absorption
2DES: two-dimensional electronic spectroscopy
TMPD: tetramethyl-*p*-phenylenediamine
GSB: ground-state bleaching
ESA: excited-state absorption
2D-DAS: 2D-decay associated spectra
