# Supplementary material for: Direct Evidence for Excitation Energy Transfer Limitations Imposed by Low-Energy Chlorophylls in Photosystem I–Light Harvesting Complex I of Land Plants
Source: J Phys Chem B. 2021 Mar 31;125(14):3566–73. doi: 10.1021/acs.jpcb.1c01498 (PMC8154617; doi:10.1021/acs.jpcb.1c01498)
Supplement: Supplementary file 1 — jp1c01498_si_001.pdf [file jp1c01498_si_001.pdf]

Direct Evidence for Excitation Energy Transfer Limitations Imposed by Low-energy Chlorophylls in the Photosystem I – Light Harvesting Complex I of Land Plants

Mattia Russo[1], Anna Paola Casazza[2], Giulio Cerullo[1], Stefano Santabarbara[3] and Margherita Maiuri[1*]*

[1] Istituto di Fotonica e Nanotecnologie del Consiglio Nazionale delle Ricerche, Dipartimento di Fisica, Politecnico di Milano, Piazza Leonardo da Vinci 32, 20133 Milano, Italy

[2] Istituto di Biologia e Biotecnologia Agraria, Consiglio Nazionale delle Ricerche, Via Bassini 15a, 20133 Milano, Italy

[3] Photosynthesis Research Unit, Centro studi sulla Biologia Cellulare e Molecolare delle Piante, Consiglio Nazionale delle Ricerche, Via Celoria 26, 20133 Milano, Italy

Supporting Information

Section I:

The overall structural organisation of chromophore arrangements in higher plants PSI-LHCI is shown in Figure S1a. Chlorophyll (Chl) *a* bound to the core complex (from PDB 4XK8[1]) are marked in golden colours. Red and blue Chls (*a* + *b*) are bound to the LHCI complement. Red complexes identify Lhca4 and Lhca3 monomers which harbour the red-most shifted transitions. Carotenoids are labelled in orange.

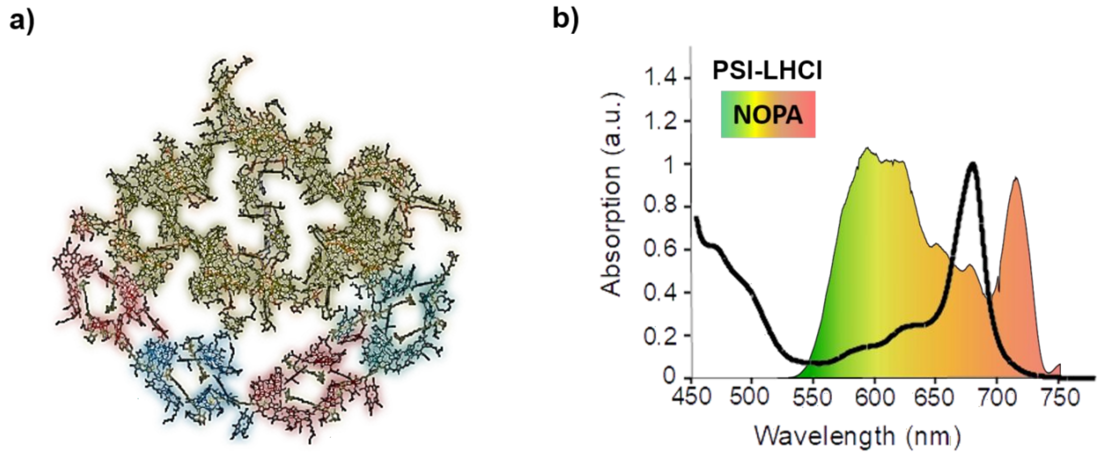

Figure S1: Panel **a** shows the structural organization of chromophores within PSI-LHCI of higher plants (view from the luminal side). Panel **b** shows the comparison of the steady state absorption spectrum of PSI-LHCI (black line) and the laser pulse spectrum (shaded line).

Section II:

2DES MAPS of PSI-LHCI at closed centers

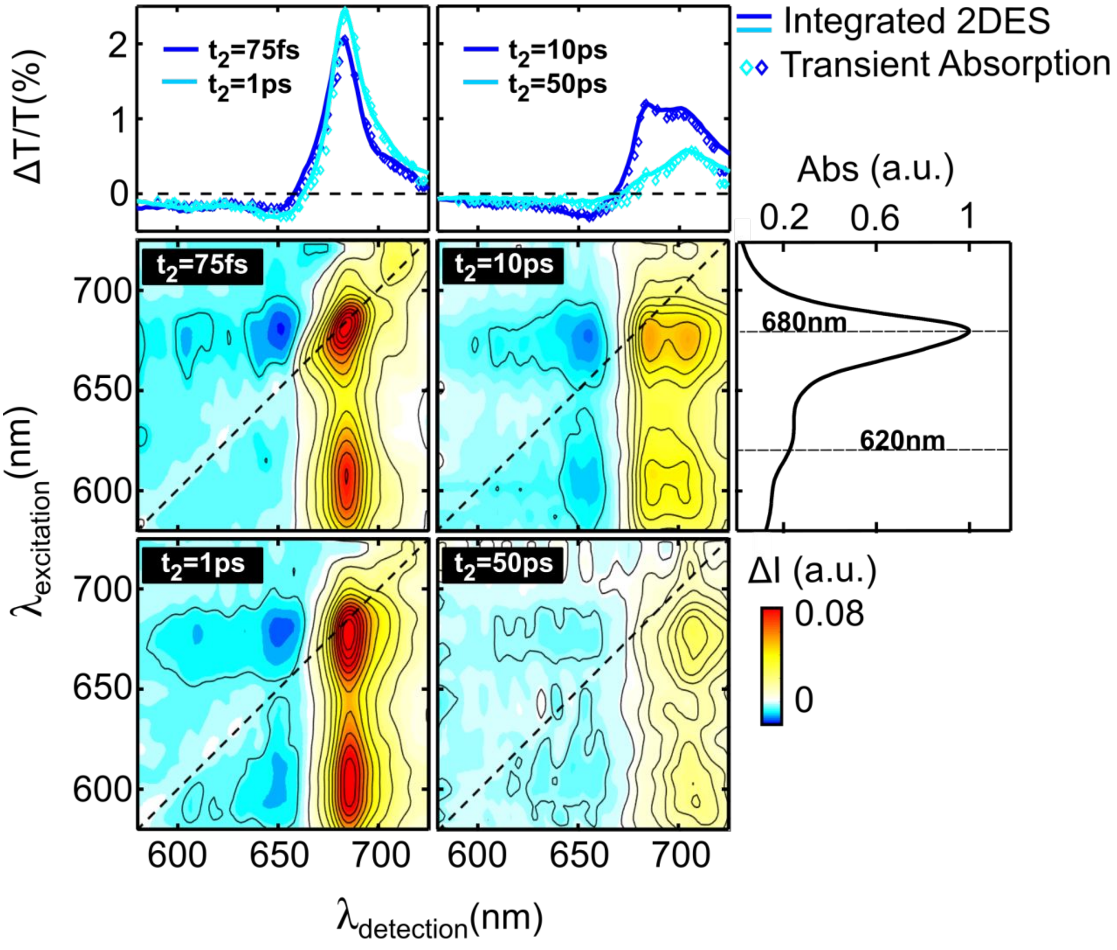

Figure S2 shows the differential absorption spectra of the PSI-LHCI supercomplex at four different waiting times: t_2 = 75 fs, 1 ps, 10 ps and 50 ps. On top: Integrated 2DES spectra along the excitation wavelength axis (continuous line) overlapped with the transient absorption spectra (symbols). On the right: portion of the steady state absorption spectrum from 580 nm to 730 nm.

Section III:

Expanded 2DES MAPS and 2D-DAS in the red forms dominated window

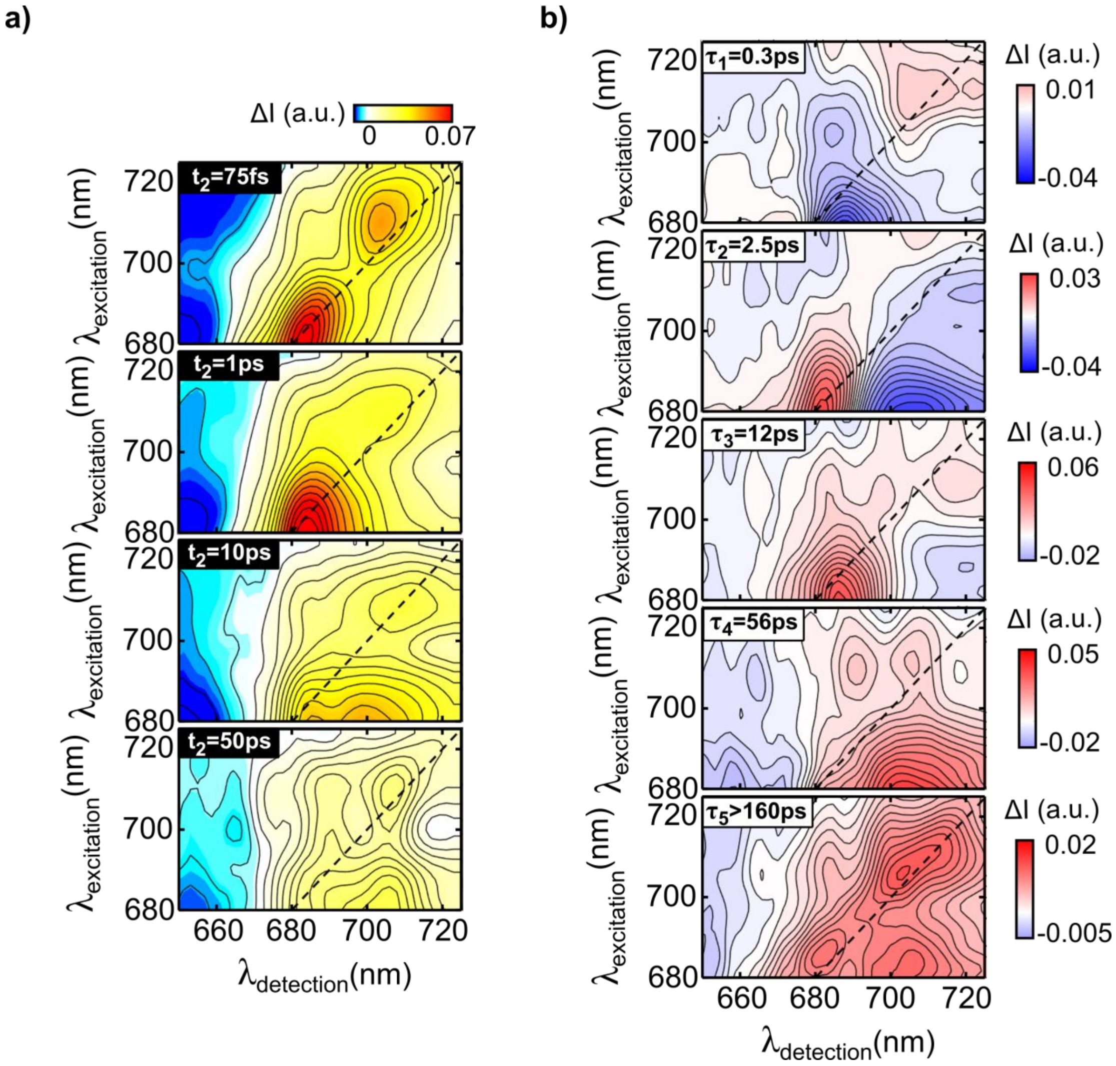

Figure S3. Panel **a:** expanded 2DES maps in the spectral window dominated by the long-wavelength antenna states of PSI-LHCI (red forms) under open centres conditions and at representative t_2 delays. Panel **b** shows the details of 2D-DAS in the spectral window of the red forms.

The expanded 2DES maps (Figure S3a) show the persistence of dynamics along the diagonal pump/probe axis, whereas most of the evolution results in an off-diagonal cross peak(s), centered, initially, at 680 and then further shifting to 700 nm for pumping in the bulk absorptions (λ_{pump}<700 nm). Figure S3b shows an expanded view of the 2D-DAS (for open centre conditions) in the red forms spectral window. It is noticeable that in the fastest DAS (0.3 ps) there is an inversion of sign, due to

prompt depopulation of the red forms by energetically uphill energy transfer to the bulk, whereas a downhill energy transfer to these states is observed for λ_{pump}<700 nm. The inversion of the amplitude sign is also present in the 3 ps 2D-DAS, indicating a slower kinetic component reflecting the pre-equilibration of the bulk absorption from the red forms. Furthermore, the persistence of depopulation dynamics along the pump/probe diagonal is also observed in this spectral window in 2D-DAS associated with the 12 ps, 56 ps and, even more clearly, in the >160 ps lifetimes. This further spectral-temporal evolution corresponds to the depopulation of red-forms having absorption maxima at ~705 and ~710 nm respectively. The progressive slowdown of de-excitation correlating with the red-shift of different long-wavelength absorption forms is in excellent agreement with previous excited state relaxation dynamics studies[2–6].

Decay associated spectra (DAS) at selected pump-wavelengths

To facilitate the comparison of the DAS obtained from the analysis of 2DES data with those obtained previously from classical TA techniques with preferential excitation at defined wavelengths, slices of the 2D-DAS map (Figure 2) are shown below for PSI-LHCI under open and closed centers conditions.

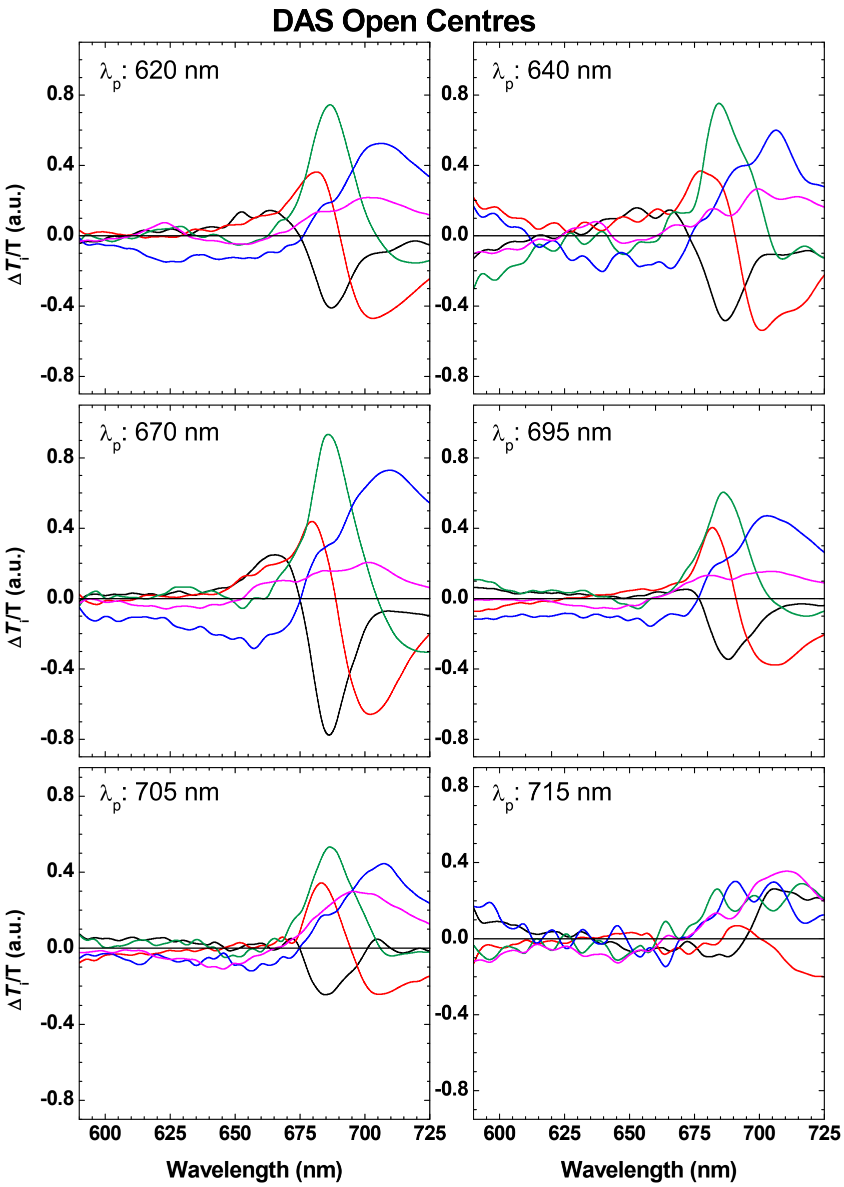

Figure S4. Decay Associated Spectra (DAS) for PSI-LHCI under open centers conditions, extracted from the 2D-DAS map of Figure 2a, at selected excitation wavelengths across the absorption spectrum. DAS were binned on a 5 nm window around the indicated pump wavelength. Black lines: 0.3 ps; Red lines: 2.5 ps; Green Lines: 12 ps; Blue lines: 56 ps: Magenta lines: >160 ps. For ease of comparison all spectra have been normalised to the maximum ΔT/T signal extrapolated at t_2=0.

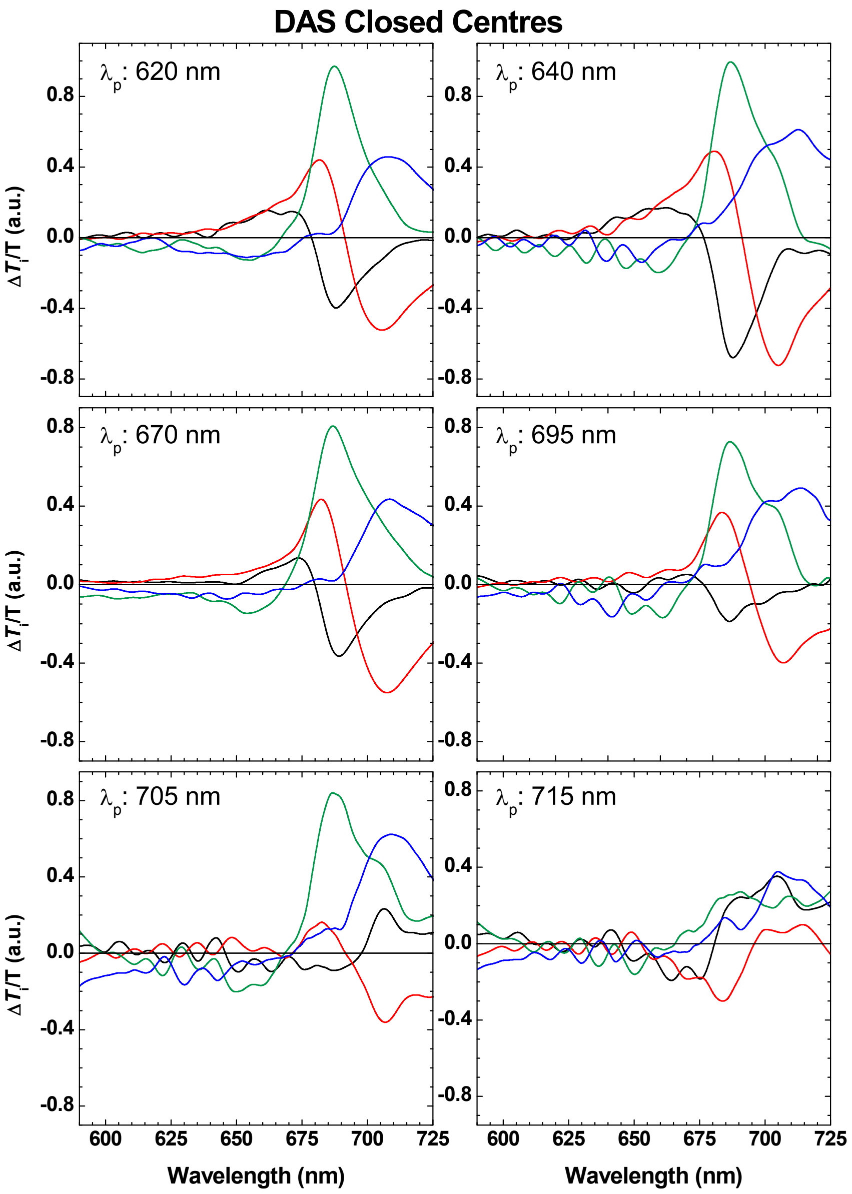

Figure S5. Decay Associated Spectra (DAS) for PSI-LHCI under closed centers conditions, extracted from the 2D-DAS map of Figure 2b, at selected excitation wavelengths across the absorption spectrum. DAS were binned on a 5 nm window around the indicated pump wavelength. Black lines: 0.2 ps; Red lines: 3.5 ps; Green Lines: 22 ps; Blue lines: 78 ps. For ease of comparison all spectra have been normalised to the maximum ΔT/T signal extrapolated at t_2=0.

Section IV: Definition of mean decay lifetime τ_m, sections at representative pump wavelengths and comparison with values retrieved from fluorescence lifetime studies.

The mean decay lifetime (τ_m) is defined as the first moment of the normalised decay distribution. When the decay function is a linear combination of exponentials, the moment has an analytical solution, given by:

$$\tau_m(\lambda_1,\lambda_2) = \frac{\sum_i A_i(\lambda_1,\lambda_2)\cdot\tau_i^2}{\sum_i A_i(\lambda_1,\lambda_2)\cdot\tau_i} \tag{1}$$

Where $A_i(\lambda_1,\lambda_2)$ are 2D-DAS, functions of λ_1 and λ_2, the probe and pump wavelength, respectively and τ_i are the lifetimes. This parameter represents the mean value of the decay. Any dependence of τ_m on either λ_1 and λ_2 indicates differences in the mean rate of excited state deactivation resulting from the presence of more rapidly/slowly equilibrating chromophore pools (observed on λ_1) whose preferential excitation is probed over λ_2.

In several previous studies, employing fluorescence detection[2–5], the probe wavelength (λ_1) dependence was explored by determining the "average fluorescence lifetime", $\tau_{av}(\lambda_1) = \sum_i A_i(\lambda_1)\cdot\tau_1 / \sum_i A_i$ since this parameter was demonstrated to represent a reliable estimator of the *effective* trapping time, upon preferential excitation on the bulk antenna[6]. This applied to the experimental conditions employed in these studies, but the physical meaning of the parameter is less straightforward for selective/preferential excitation of the RC or in general of specific pigment pools. The τ_m parameter is less strictly connected to the *effective* (photochemical) trapping when present, but is obviously dependent on it. Its generality, representing the mean value of the (normalised) decay distribution, renders it also applicable to conditions when photochemistry is not present (*e.g.* at closed centres) and/or different processes competing with natural relaxation of the excited state are present.

Figure S6 shows some slices, along λ_1, from the $\tau_m(\lambda_1,\lambda_2)$ map of Figure 4, from which the characteristic increase in mean lifetime can be observed across the entire absorption band, whereas the results in the main text focus specifically on λ_2

>680 nm. It can be appreciated that, analogously to what previously reported for τ_{av}, a smooth increase of τ_m for increasing probe wavelengths is clearly obtained for excitation (λ_2) below 700 nm, but a larger value is instead retrieved when exciting in the absorption tail at 715 nm. A noticeable difference concerns the excitation around 640 nm (within a 5 nm interval resulting from binning) in which an increase in the τ_m value comparable to the one found for the bulk absorption is observed instead, as also evident from the map shown in Figure 4a. This wavelength window corresponds to preferential excitation of Chl *b* molecules, which are located in the LHCI antenna and are therefore in close proximity to the red forms.

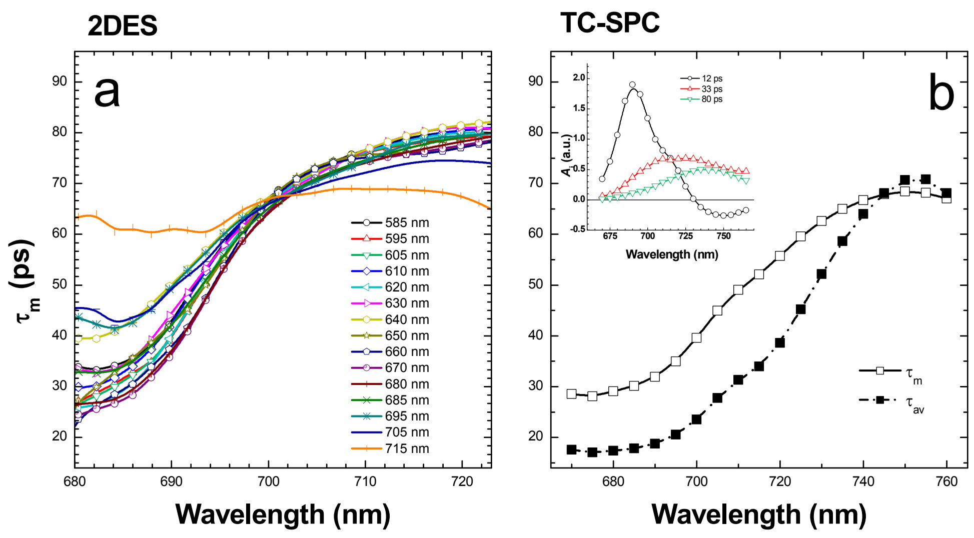

Figure S6. a: sections at selected pump wavelengths of the observation wavelength dependence of τ_m. The inversion of the trend and the increase of τ_m values in the bulk absorption region upon excitation above 700 nm is clearly observable. . τ_m slices are here binned over a 5 nm window around the indicated pump wavelength. **b**: comparison of τ_m (open symbols) and τ_{av} (closed symbols) calculated from the Decay Associated Spectra (DAS, presented in the inset) retrieved from time-correlated single photon counting (TC-SPC) analysis of PSI-LHCI, upon excitation at 632 nm[7].

We thus attribute the slowing down observed upon excitation around 640 nm to a limitation linked to excited state pre-equilibration within the external antenna, due to the presence of red forms in LHCI complexes, rather than to a direct slow transfer from Chl *b* to the bulk (Chl *a*) absorption of the core complex.

To allow comparison with previous fluorescence studies, the τ_{av} values obtained from the lifetime/DAS analysis of fluorescence of PSI isolated from spinach[7] are also presented, and compared with τ_m. Values of τ_m are generally larger than τ_{av} because the longer-lived components acquire weight due to the square power dependence in the nominator of Equation 1. Nonetheless, the trend indicating an increase in the decay distribution moment towards long emission wavelength is clear, irrespectively of the parameter considered. Although these measurements were obtained under open centers conditions, the overall relaxation dynamics change only very slightly upon P_{700} pre-oxidation, at room temperature, and no long-lived fluorescence signal is present associated to the radical pair, since their vanishingly small fluorescence yield renders them practically undetectable.

Section V: Reproducibility of the experimental results and analysis.

In the following paragraph data are presented for an independent set of measurements performed on the same PSI-LHCI preparation. The measurement conditions were the same as in the data presented in the main text, but the pulse spectrum was further shifted to the blue (down to 550 nm) and, as a consequence, had lower intensity in the near infrared above 700 nm. Moreover, t_2 kinetics were acquired only up to 40 ps. Therefore, slowly decaying component are less dynamically resolved in this accessory set of data. Nonetheless, all the main features reported in the main body of the manuscript are highly reproducible.

Open centers conditions:

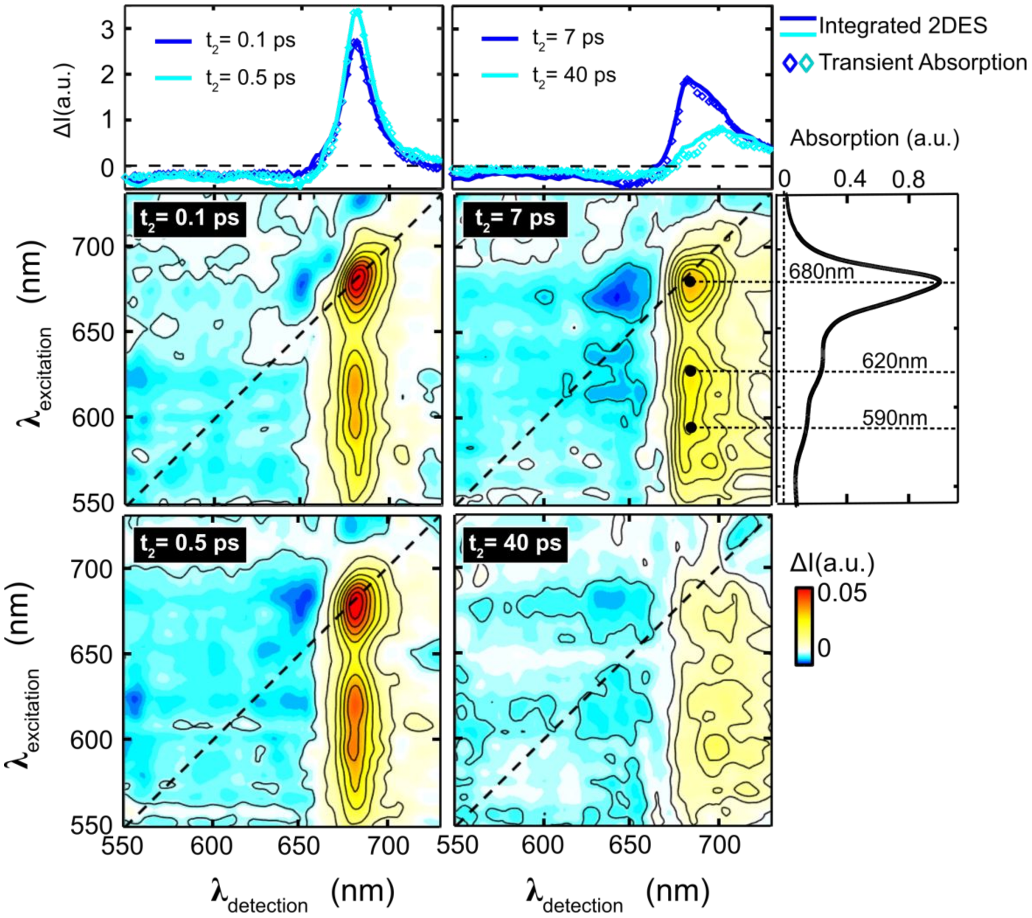

Figure S7. 2DES maps of the PSI-LHCI supercomplex under open centers conditions at four different waiting times: t_2 = 100 fs, 500 fs, 7 ps and 40 ps. On top: integrated 2DES spectra along the excitation axis (continuum line) overlapped with the TA spectra obtained by fixing to zero the coherence time (symbols). On the right: portion of the steady state absorption spectrum from 550 nm to 730 nm.

Closed centers conditions:

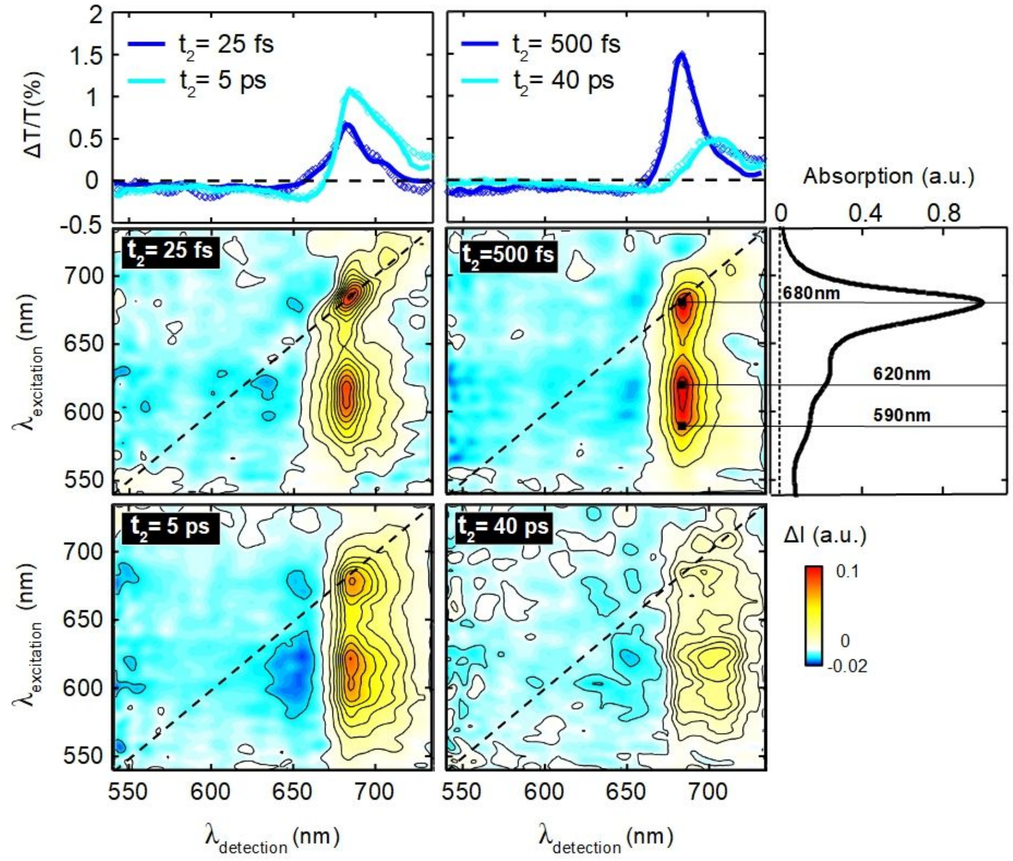

Figure S8. 2DES maps of the PSI-LHCI supercomplex under closed centers conditions at four different waiting times: t_2 = 25 fs, 500 fs, 5 ps and 40 ps. On top: Integrated 2DES spectra along the excitation axis (continuum line) overlapped with the transient absorption spectra obtained by fixing to zero the coherence time (symbols). On the right: portion of the steady state absorption spectrum from 550 nm to 730 nm.

2D-DAS resulting from the analysis of data for which representative 2DES maps are shown in Figure S7 and S8.

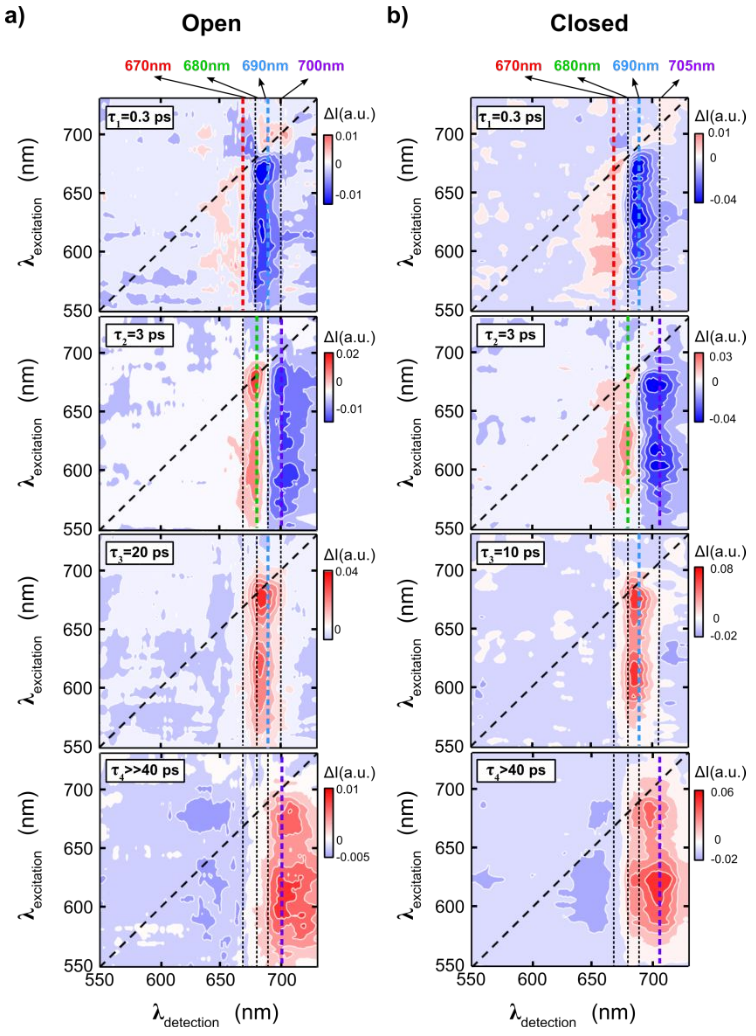

Figure S9. Global analysis results: a) 2D-DAS maps of PSI-LHCI with open RCs. The temporal evolution of the system can be described with four time constants: 0.3 ps, 3 ps, 20 ps and a non-decaying component (limited by the maximum delay acquired for the measurement); b) 2D-DAS maps of PSI-LHCI with closed RC. The temporal evolution of the system can be described with four time constants: 0.3 ps, 3 ps, 10 ps and a non-decaying component.

The 2D-DAS and the associated lifetimes obtained in an independent measurement set and employing slightly different acquisition conditions, closely resemble those reported in Figure 3 of the main text. The main difference relates to the long-lived components which are not fully resolved in Figure S9 (with respect to Figure 3) because of the shorter t_2 scale employed in these measurements.

It can nonetheless be appreciated that there are specific spectral features characterising the long-lived component (>40 ps) that are specific for open and closed centre conditions. In the first case, a broader GSB contribution is retrieved having maximum intensity at ~700 nm. The corresponding 2D-DAS spectral feature narrows and red-shifts upon centers closure. This difference is interpretable in terms of the disappearance of the long-lived signal due to P_{700}^{+} under closed centers, when only the slowly decaying excited relaxation of the low energy state is observed instead.

In the 2D-DAS reported in Figure 3 the contributions of EET from the red-forms and the long-lived (non-decaying) contribution from P_{700}^{+} are better, but not fully, separated. This is because in PSI-LHCI supercomplex of higher plants the relaxation of the low energy state has been shown to be polyphasic, often characterised by lifetimes of ~30-40 ps and 60-80 ps, respectively. The latter lifetime is clearly resolved under closed centers conditions (Figure 3 and Figure S3), but it is cumbersome to fully deconvolute it from the non-relaxing P_{700}^{+}, which is known to decay on a millisecond time scale in the isolated photosystem.

References.

(1) Qin, X.; Suga, M.; Kuang, T.; Shen, J.R. Photosynthesis. Structural basis for energy transfer pathways in the plant PSI-LHCI supercomplex. *Science* **2015**, *348*, 989–995.

(2) Engelmann, E.; Zucchelli, G.; Casazza, A.P.; Brogioli, D.; Garlaschi, F.M.; Jennings, R.C. Influence of the Photosystem I-Light Harvesting Complex I Antenna Domains on Fluorescence Decay. *Biochemistry* **2006**, *45*, 6947–6955.

(3) Galka, P.; Santabarbara, S.; Khuong, T.T.; Degand, H.; Morsomme, P.; Jennings, R.C.; Boekema, E. J.; Caffarri, S. Functional analyses of the plant Photosystem I-Light-Harvesting Complex II supercomplex reveal that Light-Harvesting Complex II loosely bound to Photosystem II is a very efficient antenna for Photosystem I in State II. *Plant Cell* **2012**, *24*, 2963–2978.

(4) Jennings, R.C.; Zucchelli, G.; Santabarbara, S. Photochemical trapping heterogeneity as a function of wavelength in plant Photosystem I (PSI-LHCI). *Biochim. Biophys. Acta* **2013**, *1827*, 779–785.

(5) Santabarbara, S.; Tibiletti, T.; Remelli, W.; Caffarri, S. Kinetics and heterogeneity of energy transfer from Light Harvesting Complex II to Photosystem I

in the supercomplex isolated from arabidopsis. *Phys. Chem. Chem. Phys.* **2017**, *19*, 9210–9222.

(6) Jennings, R.C.; Zucchelli, G.; Croce, R.; Garlaschi, F.M. The Photochemical trapping rate from red spectral states in PSI-LHCI is determined by thermal activation of energy transfer to bulk chlorophylls. *Biochim. Biophys. Acta* **2003**, *1557*, 91–98.

(7) Molotokaite, E.; Remelli, W.; Casazza, A.P.; Zucchelli, G.; Polli, D., Cerullo, G.; Santabarbara, S. Trapping dynamics in Photosystem I-Light Harvesting Complex I of higher plants is governed by the competition between excited state diffusion from low energy states and photochemical charge separation. *J Phys Chem B.* **2017,** 121, 9816–9830.
